# Retraction: Doripenem, Ertapenem, and Meropenem Sensitivity in Salmonella Typhi: A Cross-Sectional Study From Pakistan

**DOI:** 10.7759/cureus.r161

**Published:** 2025-01-16

**Authors:** Mian Mufarih Shah, Imran Khan, Mehwash Iftikhar, Nazir Shah, Saeed Ur Rahman, Jahanzeb Khan

**Affiliations:** 1 Department of Medicine, Medical Teaching Institution (MTI) Hayatabad Medical Complex, Peshawar, PAK; 2 Department of Internal Medicine, Hayatabad Medical Complex, Peshawar, PAK; 3 Department of Medicine, Hayatabad Medical Complex, Peshawar, PAK; 4 Department of Pathology, Khyber Girls Medical College, Peshawar, PAK; 5 Department of Microbiology, Hayatabad Medical Complex, Peshawar, PAK; 6 Department of Pharmacy, Hayatabad Medical Complex, Peshawar, PAK

This article has been retracted by the Editors-in-Chief due to concerns regarding scientific accuracy. The article reports high resistance rates of S. typhi to carbapenem despite insufficient supporting evidence of high rates of such resistance in Pakistan or anywhere globally.

